# The Changes of Retinal Saturation after Long-Term Tamponade with Silicone Oil

**DOI:** 10.1155/2015/713828

**Published:** 2015-10-18

**Authors:** Bingsheng Lou, Zhaohui Yuan, Liwen He, Lixia Lin, Qianying Gao, Xiaofeng Lin

**Affiliations:** State Key Laboratory of Ophthalmology, Zhongshan Ophthalmic Center, Sun Yat-Sen University, Guangzhou 510060, China

## Abstract

*Purpose.* To evaluate the effects of long-term tamponade with silicone oil on retinal saturation. *Methods.* A total of 49 eyes that received tamponade with silicone oil were included. The patients were divided into 3 groups (3–6 months, 6–9 months, and >9 months) according to the duration of silicone oil tamponade. Retinal oximetry was performed using the Oxymap system before and 2 months after silicone oil removal. *Results.* The mean retinal oxygen saturation before silicone oil removal was 107% ± 12% in the arterioles and 60% ± 10% in the venules, with an overall arteriovenous difference (AVD) of 47% ± 14%. The AVD in the >9-month group was significantly higher than that in the 3–6-month group (54% ± 16% versus 44% ± 11%, *P* = 0.042). After silicone oil removal, the AVD in the >9-month group was significantly decreased (45% ± 9% versus 54% ± 16%, *P* = 0.009); additionally, the arterioles were significantly wider than before surgery (10.8 ± 0.7 pixels versus 10.4 ± 0.9 pixels, *P* = 0.015). *Conclusions.* The tamponade with silicone oil for more than 9 months will cause the alterations of retinal saturation and the narrowing of retinal arterioles, which may further interfere with the oxygen metabolism in the retina.

## 1. Introduction

For more than three decades, the implantation of silicone oil (polydimethylsiloxane, PDMS) into the vitreous cavity following vitrectomy has been demonstrated to be effective in complicated cases of retinal detachment [1–3]. It is reported that the rate of anatomic reattachment of the retina after silicone oil tamponade is approximately 80–90% [4]. Silicone oil currently appears to be the first-choice treatment for long-term vitreous replacement [5]. However, silicone oil is still not the ideal vitreous replacement for permanent tamponade. In clinical practice, silicone oil is usually removed after 3-4 months in uncomplicated cases; however, many surgeons leave it for as long as possible in the eye, especially in complicated cases, such as severe ocular trauma and proliferative diabetic retinopathy [2, 4].

The problems of silicone oil are mainly due to the high incidence of complications of long-term tamponade, such as keratopathy, glaucoma, cataracts, and silicone oil emulsification [3]. In addition, silicone oil has the potential to cause retinal toxicity. It has been revealed that the histological and ultrastructural changes after silicone oil tamponade are primarily located in the outer plexiform layer and include the disappearance of the processes of horizontal and bipolar cells and of the synaptic terminals of the photoreceptors in enucleated human eyes [6, 7] and in experimental silicone oil-filled eyes [8]. As previously reported, the retinal histological changes mostly appeared from 6 months to 1 year after silicone oil injection [6–8]. It has been thought that impurities in silicone oil, such as low-molecular-weight components (LMWC), ionic compounds, compounds with cleavable fluoride, and residual catalysts, are responsible for the ocular toxicity [9, 10].

Apart from its potential retinal toxicity, the gravity and mechanical pressure of the silicone oil on the retina may influence retinal blood flow and, in part, may result in secondary changes in the retina [9, 11, 12]. Effert et al. found that the arteriovenous passage time was prolonged in the silicone oil-filled eyes 3–5 days after surgery by using a scanning laser ophthalmoscope after the intravenous injection of sodium fluorescein [13]. Kubicka-Trzaska et al. also found that the macular microcirculation blood flow was significantly reduced in silicone oil-filled eyes one month after surgery by using Doppler laser scanning [11]. Moreover, the presence of silicone oil in the vitreous cavity may block the oxygen exchange between the retinal surface and the vitreous humor, resulting in the metabolic disturbance of the retina [9, 12]. Because of the high oxygen consumption of the outer retina (photoreceptor), the hypoxemia of retina may be easier to cause pathological change. To date, due to the lack of available measurements to detect retinal oxygenation directly, there are little definitive data to support the effect of silicone oil tamponade on the oxygen metabolism in the retina. In 2006, Hardarson et al. introduced a noninvasive technique of retinal saturation by automatic retinal oximetry, which was considered as a highly accurate and repeatable method of assessing retinal saturation [14, 15]. In the current study, we utilized this noninvasive measurement to evaluate the effect of silicone oil tamponade on oxygen saturation and diameter in retinal vessels in traumatic retinal detachment cases.

## 2. Methods

This was a prospective pilot study designed to investigate the effect of silicone oil tamponade on retinal vessel oxygen saturation. The study protocol was approved by the medical ethics committee of the Zhongshan Ophthalmic Center at Sun Yat-Sen University in China (number 2013MEKY028), and the study was performed in accordance with the principles of the World Medical Association Declaration of Helsinki. All patients signed informed consent forms prior to enrolment.

### 2.1. Patients

The patients with silicone oil tamponade who encountered traumatic retinal detachment were recruited before silicone oil removal. According to the medical records, all subjects received tamponade with the same type of silicone oil (highly purified silicone oil, 5000 cps, RT SIL-OL 5000, Carl Zeiss Meditec AG, Jena, Germany), combined with the primary 20 G pars plana vitrectomy and laser photocoagulation. The inclusion criteria were as follows: initial silicone oil tamponade duration of more than 3 months, transparent reflecting media, intraocular pressure (IOP) between 11 and 21 mmHg, complete retinal reattachment, and healthy contralateral eye. The exclusion criteria included severe refractive media opacity (serious keratoleukoma and cataracts), silicone oil emulsification, ocular hypertension, retinal detachment, retinal scar within the main vascular arch, any ocular disease and any history of surgery in the contralateral eye, any type of systemic disease, and pregnancy. The past medical history and medications were obtained from the subjects after they had consented to participate in the study. Any recognizable silicone oil droplet in aqueous humor or on the retinal surface under slit-lamp biomicroscopy and thorough three-mirror lens fundus examination was identified as silicone oil emulsification in this study. The patients were divided into 3 groups based on the duration of silicone oil tamponade: the 3–6-month group, the 6–9-month group, and the >9-month group.

### 2.2. Surgical Procedures

All subjects underwent silicone oil removal surgery, and regional anesthesia with a peribulbar block was employed in all cases. The surgical incisions were performed by using the classic 20 G pars plana vitrectomy (PPV). Silicone oil was removed via a 20 G syringe needle with the manual vacuum. After silicone oil removal, the vitreous cavity was lavaged three times with balanced salt solution (BSS) to remove the droplets of silicone oil. The subjects were excluded from the study if they had laser photocoagulation, perfluorocarbon tamponade, buckling sclera, or epiretinal membrane peeling combined with the silicone oil removal. The surgical procedures were performed by skilled surgeons who were unaware of the duration of silicone oil tamponade and the retinal saturation of the subjects.

### 2.3. Clinical Examinations and Follow-Up

All patients underwent comprehensive clinical examinations. The examinations performed on both eyes included the following: slit-lamp microscopy, thorough three-mirror lens fundus examination, best corrected visual acuity (BCVA) measurement, noncontact tonometry, and noninvasive spectrophotometric retinal oximetry. The systemic examinations included blood pressure, heart rate measurements, and finger pulse oximetry. The baseline clinical examinations were performed 24 hours before the time of surgery. All subjects were followed up at 2 weeks, 1 month, and 2 months from previous examinations. The second retinal oximetry was repeated approximately 2 months after silicone oil removal. Subjects were excluded from the study if postoperative ocular hypertension, retinal detachment, vitreous hemorrhage, or choroidal detachment occurred.

### 2.4. Retinal Oximetry

**Figure 1:**
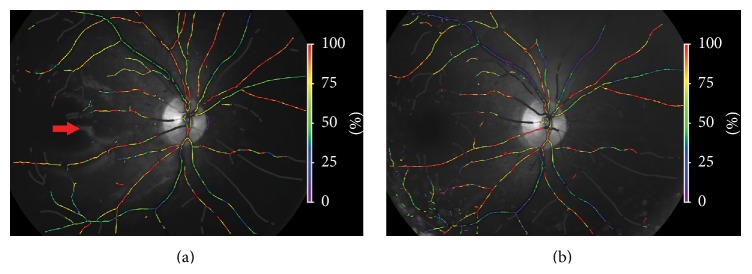
Sample images obtained from the same eye before (a) and after (b) silicone oil removal. The red arrow in the left image indicates the reflected light of the interface of the silicone oil bubble on the retinal surface. The reflected light should be prevented from appearing in the measurement zone if possible by adjusting the angle of the camera.

The automated oximeter (Oxymap, Inc., Reykjavik, Iceland) was used for retinal oximetry measurement. The Oxymap system is installed on a fundus camera (Topcon TRC-50DX; Topcon Co., Tokyo, Japan). Details regarding the device characteristics have been described previously [16]. The pupils of the subjects were dilated with 0.5% tropicamide (Shenyang Xingji Co., Shenyang, China) before retinal oximetry. All fundus images were obtained in a dark room by the same skilled technician using standard procedures and parameters as follows: (1) the lowest illumination intensity; (2) a flash intensity of 75 Ws; (3) small aperture and small pupil; (4) a consistent angle of gaze; (5) the same order of photographs (right first; two images for each eye: one image centered on the macula and one image centered on the optic disc; the highest quality image with the optic disc in the center was selected for analysis); and (6) the reflex of silicone oil on the retinal surface was prevented from appearing in the measurement zone by adjusting the angle of the camera (see Figure 1). Oxymap analysis software (Oxymap version 2.4, Oxymap, Inc.) was used to analyze the oxygen saturation (SaO_2_). Measurements from the images were analyzed by the same examiner following the standardized protocol described previously [17]. In each image, SaO_2_ was measured in all first and second branches of the retinal arterioles and venules measuring above 6 pixels in vessel diameter in the measurement zone, which extended from 20 pixels to 220 pixels from the optic disc margin (Figure 1). The arteriovenous difference was calculated by subtracting the oxygen saturation in the venules from the oxygen saturation in the arterioles. The examiner was unaware of all the clinical details of the subjects before and after surgery.

### 2.5. Statistical Analysis

All data were analyzed using SPSS version 19.0 (SPSS Inc., Chicago, IL, USA). Data are expressed as the means ± standard deviations (SD), and a *P* value < 0.05 was considered statistically significant. The normality of distribution was tested by the Shapiro-Wilk test, and two data sets (silicone oil eye and contralateral eye, before surgery and after surgery) were compared by paired *t*-tests. The subgroup analysis (subgroups divided by duration of silicone oil tamponade) was performed by one-way analysis of variance (ANOVA).

## 3. Results

The total number of recruited patients who completed the 2-month follow-up after silicone oil removal was 49. The mean age of the subjects was 38 ± 13 years, and all the subjects were of Chinese ethnicity. After silicone oil removal, there was one case of ocular hypertension and one case of retinal detachment, and these patients were excluded from the study. The baseline characteristics are summarized in Table 1.

### 3.1. Oxygen Saturation

Mean retinal oxygen saturation for all patients before silicone oil removal (*n* = 49) was 107% ± 12% in the arterioles and 60% ± 10% in the venules, with an arteriovenous difference (AVD) of 47% ± 14%. The total oxygen saturation of the arterioles and the AVD in the silicone oil-filled eyes were higher than those in the contralateral healthy eyes (the contralateral arterial saturation was 92% ± 5%, *P* < 0.001, and the contralateral AVD was 32% ± 7%, *P* < 0.001, paired *t*-test). However, there was no significant difference in the total venous saturation between silicone oil-filled eyes and contralateral healthy eyes (the contralateral venous saturation was 61% ± 6%, *P* = 0.779, paired *t*-test) (see Table 2). After silicone oil removal, the overall AVD was significantly lower than that before surgery (the postsurgery AVD was 43% ± 8%, *P* = 0.014, paired *t*-test). However, there were no significant changes in the arterial and venous saturations before and after surgery (the postsurgery arterial saturation was 105% ± 9%, *P* = 0.061, and the postsurgery venous saturation was 62% ± 8%, *P* = 0.275, paired *t*-test) (see Table 3). Representative images from a patient taken before and after silicone oil removal are shown in Figure 1.

Analysis of the subgroups was performed according to the duration of the silicone oil tamponade, and there were no significant differences in the arterial oxygen saturations and venous oxygen saturations between the groups (see Table 2). However, we did find a statistically significant difference in the AVD between the 3–6-month and >9-month groups (44% ± 11% versus 54% ± 16%, resp., *P* = 0.042, one-way ANOVA) (see Table 2). We further compared the retinal oxygen saturation before and after silicone oil removal in each group. Interestingly, only in the >9-month group, we did find a significant difference in the retinal oxygen saturation before surgery and after surgery (a decreased AVD: 54% ± 16% before surgery versus 45% ± 9% after surgery, resp., *P* = 0.009, paired *t*-test). However, in the 3–6-month and 6–9-month groups, there were no statistical changes in the retinal oxygen saturation after silicone oil removal (see Table 3).

### 3.2. Width of Retinal Vessel

The mean diameter of the retinal vessels in all patients before silicone oil removal was 11.0 ± 1.0 pixels in the arterioles and 14.2 ± 1.4 pixels in the venules. In the >9-month group, the arterioles were significantly thinner than those in the contralateral eye (10.4 ± 0.9 pixels versus 11.8 ± 0.6 pixels, resp., *P* < 0.001, paired *t*-test) (Table 4). There was no significant difference in the venules in the silicone oil-filled eye compared with the contralateral eye. Additionally, we compared the width of the retinal vessels before and after silicone oil removal. In the >9-month group, the arterioles were significantly wider than before surgery (10.8 ± 0.7 pixels versus 10.4 ± 0.9 pixels, *P* = 0.015, paired *t*-test). However, there were no significant differences in the arteriole widths in the other groups before and after silicone oil removal (Table 5).

## 4. Discussion

In this prospective pilot study, we evaluated the effect of silicone oil tamponade on retinal oxygen saturation in subjects treated for traumatic retinal detachment. Our data demonstrated that the total mean retinal arterial oxygen saturation and AVD were increased after silicone oil tamponade, compared with the contralateral healthy eye. To minimize the effects of initial traumatic retinopathies on retinal saturation, further alterations of retinal saturation after silicone oil removal were observed. Our results showed slightly decreased arterial saturation and AVD (but no statistically significant difference) following silicone oil removal in the 3–6-month and 6–9-month groups. It can be inferred that the effects of silicone oil on retinal saturation are negligible when silicone oil is performed for less than 9 months. Therefore, the increased arterial saturation and AVD before silicone oil removal are thought to be attributed to the initial damage due to the ocular trauma and the effect of the pars plana vitrectomy. How the ocular trauma influences retinal saturation has not been well understood until now. Nevertheless, similarly increased arterial saturation was found in branch retinal vein occlusion [18] and in diabetic retinopathy [19, 20]. Increased saturations in the arterioles and/or in the venules were also found after vitrectomy in previous studies [21, 22].

However, the interpretation of the alteration of retinal saturation in the >9-month group is more complicated. Our data showed that the retinal AVD was significantly increased in the >9-month group compared with that in the 3–6-month group. As discussed above, there is no obvious direct effect on retinal saturation after silicone oil tamponade lasting 3–6 months. Does this result indicate that the increased AVD also resulted from the initial traumatic retinopathy? It is reasonable that the patients with more severe trauma usually underwent longer silicone oil tamponade. However, the increased AVD was partly recovered after silicone oil removal. Obviously, it is impossible that the effect of initial traumatic retinopathy on retinal AVD would disappear after silicone oil removal. Therefore, our results indicate that the effect of the silicone oil tamponade when it is in place for more than 9 months is partly responsible for the increased AVD. Why did the silicone oil barely influence the change in retinal saturation after tamponade of <9 months but cause the significant alteration in AVD after tamponade for more than 9 months? As described above, the retinal pathological changes usually and mostly appeared from 6 months to 1 year after silicone oil injection [6–8]. Therefore, we speculate that the effect of silicone oil tamponade for more than 9 months on retinal saturation resulted from the retinal injury due to the toxicity of silicone oil. The slight alteration in retinal saturation after silicone oil tamponade for less than 9 months can be attributed to the primary effects of the physical characteristics of silicone oil, such as the mechanical pressure exerted by the oil on the retinal surface and its blockage of metabolic exchange, but not to the retinal pathological changes due to silicone oil tamponade. It is therefore clear that the direct physical effect of silicone oil on retinal saturation is negligible. This conclusion is coincident with that of another study, which demonstrated that, after silicone oil tamponade for 6 months, the retinal vascular morphology did not display any distinct abnormalities and the hypoxia-induced factor-1 alpha (HIF-1*α*) and vascular endothelial growth factor (VEGF) concentrations did not vary markedly [23]. In addition, the partially recovered AVD after silicone oil removal may indicate the partial reversibility of the retinal pathological changes due to the silicone oil if the silicone oil is removed in time. However, in the current study, the BCVA was not significantly improved after silicone oil removal.

Moreover, the changes in retinal vessel diameters after silicone oil tamponade further support our deduction above. The diameter of retinal vessels after silicone oil tamponade for <9 months was not changed markedly. However, the diameter of the retinal arterioles was significantly narrowed after tamponade for more than 9 months, compared with the contralateral healthy eye. The diameter of the retinal arterioles was partly widened again after silicone oil removal, which was similar to the alteration seen in the AVD. This result could indicate that the long-term silicone oil tamponade is responsible for the narrowing of retinal arterioles. Relevant clinical cases reporting fluorescent angiographic findings in patients who underwent injection of silicone oil for more than one year revealed that the arterioles appeared to be narrow and occluded, with an extensive capillary nonperfusion area [24]. In this paper, the authors explained that the changes in the retinal arterioles could be due to the toxicity of silicone oil to the retinal microvasculature itself, could be secondary to the damage to the neuroretina, or could be in response to high intraretinal partial pressures of oxygen, which would be secondary to the blockage of oxygen diffusion into the vitreous cavity by the silicone oil [24]. An alternative assumption is that silicone droplets had filtered into the retinal arterioles and mechanically obstructed them, which is supported by the finding of silicone droplets lying within a retinal arteriole in an experimental study [25].

The parallel alterations of retinal arteriole diameter and AVD may help to explain how silicone oil causes changes in retinal saturation. The arteriovenous difference in retinal saturation indicates the total oxygen consumption of the retinal tissue per unit time. The oxygen demand of the tissue and the retinal blood flow per unit time may decide the value of AVD. A decreased AVD should be related to the decreased oxygen demand of the retinal tissue and/or the increased retinal blood flow per unit time. However, an increased AVD found in silicone oil-filled eye for more than 9 months may be interpreted by the increased oxygen demand of the retinal tissue and the decreased retinal blood flow per unit time. Because the traumatic retinal detachment and surgery inevitably caused the loss of retinal cells and the dysfunction of retina, the oxygen demand of reattached retina should be decreased rather than increased. Therefore, the increased AVD in this study is more likely to be resulting from the decreased retinal blood flow per unit time. Usually, the decreased retinal blood flow is combined with the decreased retinal arterial diameter. Therefore, we assume that the narrowed retinal arteriole might be responsible for the increased AVD in silicone oil-filled eye for more than 9 months. The mechanism of narrowed retinal arteriole with long-term silicone oil tamponade needs further study in the future.

Our study had some limitations. The lack of uniformity in the severity of initial traumatic retinopathies before silicone oil injection increases the difficulty to interpret the effects of silicone oil tamponade on retinal saturation. However, we have mainly focused on comparing the retinal saturation and the width of retinal vessels before and after silicone oil removal to minimize the influence of initial traumatic retinopathies on the retinal saturation. Secondly, the Oxymap system has limitation for retinal saturation measurement. The value of retinal saturation measured by the Oxymap system is only an estimate of the true oxygen saturation. Sometimes, the values of arterial saturation in our study exceeded 100%. As previously described, the calibration coefficients used in the Oxymap system derived from predominantly Caucasian subjects [26] and the difference of fundus pigmentation between Chinese descent and Caucasian descent could have contributed to the bias [21]. Since the relationship between the optical density ratio and the actual calculated value of oxygen saturation is linear [27, 28], this bias of the oxygen saturation reading would not have affected the comparisons made in our study. Additionally, the presence of severe refractive media opacity may affect the accuracy of the oxygen saturation measurement. In this study, we have excluded the cases with serious keratoleukoma, cataract, and silicone oil emulsification to minimize the error of measurement.

## 5. Conclusions

Silicone oil application for retinal detachment is mainly served by its surface tension effect and its flotation force on the retinal surface. Our study may suggest that the direct effect of the physical characteristics of silicone oil on the retinal saturation is negligible. However, the long-term tamponade with silicone oil for more than 9 months causes the increased AVD and the narrowed retinal arterioles. We hypothesize that the toxicity of silicone oil and/or the secondary changes due to the mechanical pressure of silicone oil may be responsible for the alteration of retinal saturation. Further studies are needed to elucidate the mechanism of how long-term silicone oil tamponade affects oxygen metabolism in the retina.

## Figures and Tables

**Table 1:** Clinical and demographic distribution of the patients (before silicone oil removal).

Group	Value
Total number of patients with silicone oil tamponade	49
Number of patients with aphakia	36
Number of patients with a mild cataract	8
Number of patients with a transparent lens	5
Age (mean ± SD, years)	38 ± 13
Gender	47 males, 2 females
Laterality of eye	21 right eyes, 28 left eyes
Intraocular pressure (mean ± SD, mmHg)	14.6 ± 4.2
Systolic blood pressure (mean ± SD, mmHg)	130 ± 12
Diastolic blood pressure (mean ± SD, mmHg)	79 ± 8
Duration of silicone oil tamponade (mean ± SD, months)	8.1 ± 5.4
3–6-month group	4.1 ± 0.9
6–9-month group	7.1 ± 0.7
>9-month group	14.5 ± 5.6

**Table 2:** Retinal saturation in silicone oil-filled eyes and contralateral eyes before silicone oil removal.

Duration	Arterioles			Venules			AV difference		
	Sat O_2_ (%)			Sat O_2_ (%)			Sat O_2_ (%)		
	SiO^†^	Contralateral	*P* value^*∗*^	SiO	Contralateral	*P* value^*∗*^	SiO	Contralateral	*P* value^*∗*^
3–6 months (*n* = 18)	106 ± 9	93 ± 6	<0.001	62 ± 9	60 ± 6	0.548	44 ± 11^*∗∗*^	33 ± 6	0.001
6–9 months (*n* = 16)	105 ± 12	92 ± 5	0.002	60 ± 11	60 ± 7	0.930	44 ± 13	32 ± 7	0.006
>9 months (*n* = 15)	111 ± 14	91 ± 5	<0.001	58 ± 9	62 ± 6	0.157	54 ± 16^*∗∗*^	30 ± 6	<0.001
Total (*n* = 49)	107 ± 12	92 ± 5	<0.001	60 ± 10	61 ± 6	0.779	47 ± 14	32 ± 7	<0.001

^†^SiO = silicone oil-filled eyes.

^*∗*^Compared by paired *t*-test.

^*∗∗*^The AVD between the 3–6-month and >9-month groups compared by one-way ANOVA, *P* = 0.042.

**Table 3:** Differences in the retinal saturations before and 2 months after silicone oil removal.

Duration	Arterioles			Venules			AV difference		
	Sat O_2_ (%)			Sat O_2_ (%)			Sat O_2_ (%)		
	Before surgery	After surgery	*P* value^*∗*^	Before surgery	After surgery	*P* value^*∗*^	Before surgery	After surgery	*P* value^*∗*^
3–6 months (*n* = 18)	106 ± 9	105 ± 8	0.425	62 ± 9	62 ± 6	0.934	44 ± 11	43 ± 7	0.515
6–9 months (*n* = 16)	105 ± 12	103 ± 9	0.294	60 ± 11	60 ± 11	0.883	44 ± 13	43 ± 9	0.506
>9 months (*n* = 15)	111 ± 14	107 ± 10	0.200	58 ± 9	62 ± 7	0.114	54 ± 16	45 ± 9	0.009
Total (*n* = 49)	107 ± 12	105 ± 9	0.061	60 ± 10	62 ± 8	0.275	47 ± 14	43 ± 8	0.014

^*∗*^Compared by paired *t*-test.

**Table 4:** The diameter of the retinal vessels in silicone oil-filled eyes and contralateral eyes before silicone oil removal.

Duration	Arterioles			Venules		
	Width (pixels)			Width (pixels)		
	SiO^†^	Contralateral	*P* value^*∗*^	SiO	Contralateral	*P* value^*∗*^
3–6 months (*n* = 18)	11.3 ± 1.1	11.7 ± 0.7	0.102	14.3 ± 1.6	14.3 ± 0.7	0.975
6–9 months (*n* = 16)	11.3 ± 0.8	11.7 ± 0.6	0.067	14.1 ± 1.4	14.2 ± 0.7	0.767
>9 months (*n* = 15)	10.4 ± 0.9	11.8 ± 0.6	<0.001	14.1 ± 1.1	14.4 ± 0.8	0.390
Total (*n* = 49)	11.0 ± 1.0	11.7 ± 0.6	<0.001	14.2 ± 1.4	14.3 ± 0.7	0.516

^†^SiO = silicone oil-filled eyes.

^*∗*^Compared by paired *t*-test.

**Table 5:** Differences in retinal vessel diameter before and 2 months after silicone oil removal.

Duration	Arterioles			Venules		
	Width (pixels)			Width (pixels)		
	Before surgery	After surgery	*P* value^*∗*^	Before surgery	After surgery	*P* value^*∗*^
3–6 months (*n* = 18)	11.3 ± 1.1	11.2 ± 0.7	0.893	14.3 ± 1.6	14.4 ± 1.8	0.928
6–9 months (*n* = 16)	11.3 ± 0.8	11.2 ± 0.9	0.599	14.1 ± 1.4	13.9 ± 0.8	0.561
>9 months (*n* = 15)	10.4 ± 0.9	10.8 ± 0.7	0.015	14.1 ± 1.1	14.0 ± 1.1	0.770
Total (*n* = 49)	11.0 ± 1.0	11.1 ± 0.7	0.366	14.2 ± 1.4	14.1 ± 1.3	0.716

^*∗*^Compared by paired *t*-test.
